# Celecoxib enhances anticancer effect of cisplatin and induces anoikis in osteosarcoma via PI3K/Akt pathway

**DOI:** 10.1186/s12935-016-0378-2

**Published:** 2017-01-03

**Authors:** Bing Liu, Shigui Yan, Liyan Qu, Jian Zhu

**Affiliations:** 1 Department of Orthopedics, 2nd Affiliated Hospital, School of Medicine, Zhejiang University, #88 Jie Fang Road, Hangzhou, 310009 Zhejiang People’s Republic of China; 2 Clinical Laboratory Centre, 2nd Affiliated Hospital, School of Medicine, Zhejiang University, #88 Jie Fang Road, Hangzhou, 310009 Zhejiang People’s Republic of China; 3 Clinical Laboratory Centre, Binjiang Hospital of Hangzhou, Hangzhou, Zhejiang People’s Republic of China

**Keywords:** COX-2, MDR, Anoikis, PI3K/Akt, Osteosarcoma

## Abstract

**Background:**

COX-2, an inducible enzyme, is associated with inflammatory diseases and carcinogenesis. Overexpression of COX-2 occurs in many human malignancies, including osteosarcoma. COX-2 positivity is form 67 to 92% in osteosarcoma, and COX-2 expresses 141-fold more in cancer stem cell spheres than daughter adherent cells. In our study, we have reported that celecoxib, a cyclooxygenase-2 inhibitor, induces apoptosis in human osteosarcoma cell line MG-63 via down-regulation of PI3K/Akt. It has been confirmed that celecoxib enhances apoptosis and cytotoxic effect of cisplatin, although the mechanism remains unclear.

**Methods:**

We have attempted to identify the anti-proliferation of celecoxib, a selective COX-2 inhibitor, and the combination of celecoxib and cisplatin in MG-63 cells, and to explore the potential molecular mechanisms involved. MG-63 cells were treated with the combination of celecoxib and cisplatin or either agent alone for 48 h in serum-supplemented medium.

**Results:**

MDR1, MRP1, BCRP and Trkb, E-cadherin, β-catenin were significantly downregulated in cells treated with the combination of celecoxib and cisplatin, and decreased β-catenin level was found in cells with wortmannin, a specific PI3K inhibitor.

**Conclusion:**

Therefore, celecoxib enhances anticancer effect of cisplatin and induces anoikis in osteosarcoma, which may be PI3K/Akt-dependent, and MDR and β-catenin-related. PI3K may be at the center of the celecoxib effects, which play an essential role in the regulation of MDR and anoikis.

## Background

The cyclooxygenase (COX) isoenzymes, known as prostaglandin (PG) rate-limiting synthase. COX-2, an inducible enzyme, is associated with inflammatory diseases and carcinogenesis, which is suspected to promote angiogenesis and tissue invasion of tumours [1, 2] and the overexpression of COX-2 has been mentioned in connection with resistance to apoptosis [3, 4]. Nonsteroidal anti-inflammatory drugs (NSAIDs) have been shown to induce apoptosis as well as potentiate the effect of chemotherapeutic agents including cisplatin [5, 6]. Celecoxib is a NSAIDs that is a specific inhibitor of COX-2. Although the precise mechanisms for the chemopreventive effects of celecoxib are not yet known, the ability of the inhibition of cell proliferation, and induction of apoptosis has been well-known [7, 8]. COX-2 is also involved in drug resistance and poor prognosis of tumor [9].

Osteosarcoma is the most common primary bone tumor in children and young adults, which has been reported to express COX-2 constitutively [10–12]. COX-2 positivity is form 67 to 92% in osteosarcoma [13, 14], and COX-2 expresses 141-fold more in cancer stem cell (CSC) spheres than daughter adherent cells [15]. Therefore, we can exert the drug to affect the over-expressed genes and achieve the therapies of human malignancies.

In our study, we have reported that celecoxib, a cyclooxygenase-2 inhibitor, induces apoptosis in human osteosarcoma cell line MG-63 via down-regulation of PI3K/Akt [16]. It has been confirmed that celecoxib enhances the apoptosis induction and cytotoxic effect of cisplatin, although the mechanism remains unclear. COX-inhibitors may sensitize cancer cells to chemotherapeutic drugs via inhibiting P-gp, MRP1 and BCRP, and enhance the effect of anticancer drugs. PI3K/Akt plays an essential role in the cell/extracellar matrix (ECM) and cell/cell adhesion. Lack of the correct adhesion, the adhesion-dependent signals will be interrupted, which will result in adhesion-related apoptosis: anoikis. We have attempted to identify the anti-proliferation of celecoxib, a selective COX-2 inhibitor, and the combination of celecoxib and cisplatin in MG-63 cells, and to explore the potential molecular mechanisms involved.

## Methods

### Reagents

Celecoxib was generously provided by Pharmacia Cor. (New York, NY, USA) and dissolved in DMSO (40 mM) as a stock solution in 4 °C. Cisplatin was purchased from Qilu Pharmaceutical Co., Ltd. (ShanDong province, China) and dissolved in normal saline (2 mg/ml) as a stock solution in 4 °C. Celecoxib and cisplatin were added at various concentrations to cells in 10% fetal bovine serum (FBS)-containing DMEM. The final DMSO concentration never exceeded 0.1% (v/v) after addition to medium. Mouse monoclonal antibodies specific for PI3K p110 (sc-8010), β-catenin (sc-7963); rabbit polyclonal antibodies specific for E-cadherin (sc-7870), Trkb (sc-8316), β-actin (sc-1616R);secondary antibodies were obtained from Santa Cruz Biotechnology, Inc.(Santa Cruz, CA, USA) were purchased from Calbiochem (San Diego, CA, USA).

### Cells and cell culture

The human OS cell line MG-63 used in this study were obtained from American Type Culture Collection (Manassas, VA), and the U2OS cells were purchased from the ATCC (HTB-96; Rockville, Maryland, USA), HOS (CRL-1547TM, ATCC) was obtained from Cell Bank of Shanghai Institute of Biochemistry & Cell Biology, Chinese Academy of Sciences (Shanghai, China). MG-63 Cells were grown in DMEM medium (Gibco Life Sciences) supplemented with 10% (v/v) heat activated fetal bovine serum (Gibco Life Sciences) in a humidified atmosphere of 5% CO_2_ at 37 °C. U2OS cells were grown in RPMI1640 medium and HOS cells were grown in Eagle’s Minimum Essential medium containing supplements as above.

### Real time quantitative RT-PCR analysis

RNA was isolated from the cells using Trizol (Invitrogen) according to the manufacturer’s instructions. cDNA was generated from total denatured RNA (2 µg) by using 1 µl oligo(dT)_18_ primer, 25 units RNase inhibitor, 2 µl dNTPs (10 mM), and a Moloney Murine Leukemia Virus reverse transcriptase cDNA synthesis kit (Promega, Madison, WI, USA). Quantitative real-time PCR was performed using Applied Biosystems 7500 Real-Time PCR System and SYBR® Premix Ex Taq™ kit (Perfect Real Time) (TaKaRa, Dalian, China). Human 18s rRNA was used as internal control. The Cycle threshold (CT) values for each gene were corrected using the mean CT value. Real-time PCR data were quantified using the ΔCT method with the formula: n = 100 × 2^−(ΔCT targeted gene − ΔCT 18s rRNA)^. Targeted genes were amplified by real time PCR with the following primers: 18s rRNA forward: 5′GACTCAACACGGGAAACCTCAC3′ and reverse: 5′CCAGACAAATCGCTCCACCAAC3′; Human BCRP, forward 5′GCAGCAGGTCAGAGTGTGGTTT3′, reverse 5′GCTGCAAAGCCGTAAATCCATA3′; Human MRP1 forward: 5′CGCTGAGTTCCTGCGTACCTAT′ and reverse: 5′CCATTCTCCATTTGCTTTGCTT3′, Human MDR1 forward: 5′CCGTGGGGCAAGTCAGTTCAT3′ and reverse: 5′CCTTCCAATGTGTTCGGCATTAG3′.

### Western blot analysis

After the exposure for 48 h, floating and adherent cells were harvested and rinsed twice with cold PBS and lysed in lysis buffer [50 mM Tris (pH 7.4), 400 mM NaCl, 50 mM NaF, 30 mM sodium PP_i_, 1 mM sodium pyrovendidate, 1% SDS, and 0.5% NP40]. Protein concentrations were determined with bicinchoninic acid protein assay (Pierce, Rockford, IL, USA). Cellular proteins, at a concentration of 40 µg, were fractionated on 12% SDS-PAGE (Invitrogen/Novex, Carlsbad, CA, USA). Proteins were then transferred to a polyvinylidene difluoride membrane (Immobilon-P; Millipore, Bedford, MA, USA). The membrane was blocked with 5% nonfat milk in TBST and incubated overnight with antibody at 4 °C. After washing three times with TBST, the membrane was incubated at room temperature for 1 h with horseradish peroxidase-conjugated secondary antibody diluted with TBST (1:1000). The detected protein signals were visualized by an enhanced chemiluminescence reaction system (Amersham, Arlington Heights, IL, USA), and the signal was visualized with X-ray film (Hyperfilm; Amersham).

### Electrophoretic mobility shift assay (EMSA) for β-catenin activation

Cells were treated with celecoxib (50 and 100 µmol/l), cisplatin (10 µg/ml), and the combined administration for 48 h. Nuclear proteins were prepared using nuclear and cytoplasmic protein extraction reagents according to the manufacturer’s protocols (Pierce, Rockford, IL, USA). The crude nuclear pellet was suspended in 200 µL buffer B (20 mmol/l HEPES pH 7.9; 25% glycerol, 1.5 mmol/l MgCl_2_; 420 mmol/l NaCl, 0.5 mmol/l DTT; 0.2 mmol/l EDTA; 0.5 mmol/l PMSF; and 4 µmol/l leupeptidin) and incubated on ice for 30 min. The suspension was centrifuged at 16,000×*g* at 4 °C for 30 min. The supernatant was collected as nuclear proteins. A non-radioactive EMSA was performed using an EMSA kit (Pierce, Rockford, IL, USA) according to the manufacturer’s instructions. Nuclear protein (4 µg) extracted from the cells or tissues was incubated with biotinylated oligonucleotides containing the β-catenin site for 30 min at room temperature. The samples were separated in a nondenaturing polyacrylamide gel (6%, with 2.5% glycerol) and blotted on a Biodyne B (0.45 mm) positively charged nylon membrane (Pall Schweiz AG, Basel, Switzerland). The biotin was labeled with alkaline phosphatase-conjugated streptavidin and alkaline phosphatase was detected with Enhanced Chemiluminescense detection system (Santa Cruz, CA, USA).

### Statistical analysis

The entire experiment was done in triplicate and the data are presented as mean ± SD. The statistical significance of differences was determined by Student’s two-tailed *t* test in two groups and one-way ANOVA in multiple groups. *P* < 0.05 was considered statistically significant. All data were analyzed with SPSS 16.0 software.

## Results

### Down-regulation of MDR1, MRP1 and BCRP correlated with increased apoptosis

**Fig. 1:**
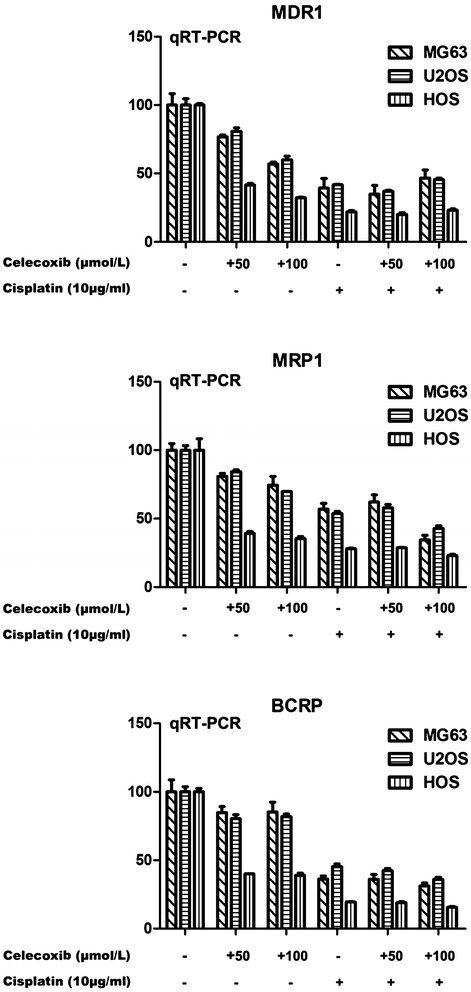
Regulation of MDR1, MRP1 and BCRP expression. RT-PCR analysis MDR1, MRP1 and BCRP expression of MG-63 cells untreated or treated with celecoxib (50, 100 µmol/l), cisplatin (10 µg/ml), and the combination for 48 h. Significant down-regulation of MDR1, MRP1 and BCRP expression was observed in cells treated with cisplatin

**Fig. 2:**
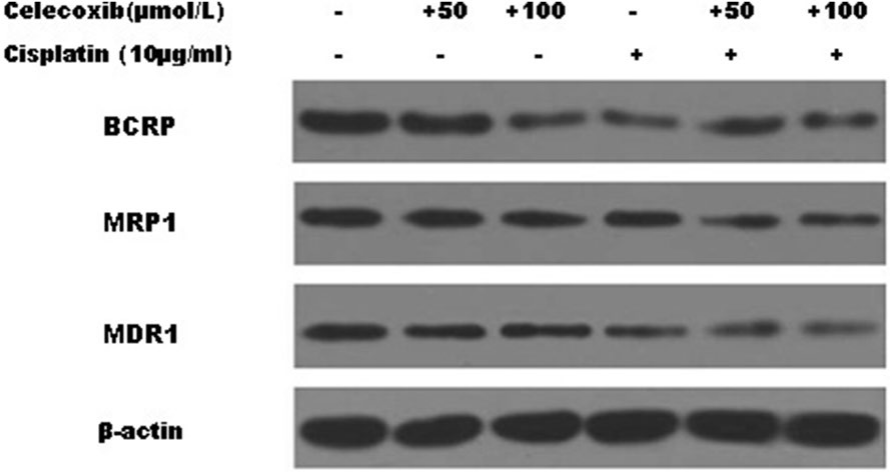
Regulation of MDR1, MRP1 and BCRP expression. Western blot analysis MDR1, MRP1 and BCRP expression of MG-63 cells untreated or treated with celecoxib (50, 100 µmol/l), cisplatin (10 µg/ml), and the combination for 48 h. Significant down-regulation of MDR1, MRP1 and BCRP expression was observed in cells treated with cisplatin

We have reported that celecoxib caused G1 phase arrest and significantly inhibited cell growth, as well as potentiating cisplatin-induced apoptosis. MG-63 cells were exposed to celecoxib (50 and 100 µmol/l), cisplatin (10 µg/ml), and the combined administration for 48 h, and the apoptosis rate was 1.39, 4.06, 5.98, 5.93, 6.66, 37.15%, respectively, which may be result from COX-2-related drug resistance. The activity of COX-2-PGE2-Prostaglandin E Receptors signal pathway can upregulate the expression of all three ABC transporters, MDR1/P-gp (multidrug resistance/P-glycoprotein), MRP1 (multidrug resistance protein 1) and BCRP (breast cancer resistance protein), which encode efflux pumps, play important roles in the development of multidrug resistance [17]. The potential mechanism of MDR1, MRP1 and BCRP in celecoxib and/or cisplatin treated cells were therefore investigated. Figure 1 shows that the downregulation of MDR1, MRP1 and BCRP mRNA expression was found in cells treated with celecoxib (50 and 100 µmol/l), cisplatin (10 µg/ml), and the combined administration for 48 h. Moreover, significant downregulation of MDR1, MRP1 and BCRP was detected in cells treated with the combination of celecoxib (100 µmol/l) and cisplatin compared with either agent alone, with apoptosis being strongly increased. However, no potentiation of celecoxib (50 µmol/l) and cisplatin was found. The results of treated MG-63 cells were similar in Western blot analysis (Fig. 2).

### Modulation of Trkb, E-cadherin and β-catenin expression in MG-63 cells treated with celecoxib and cisplatin

**Fig. 3:**
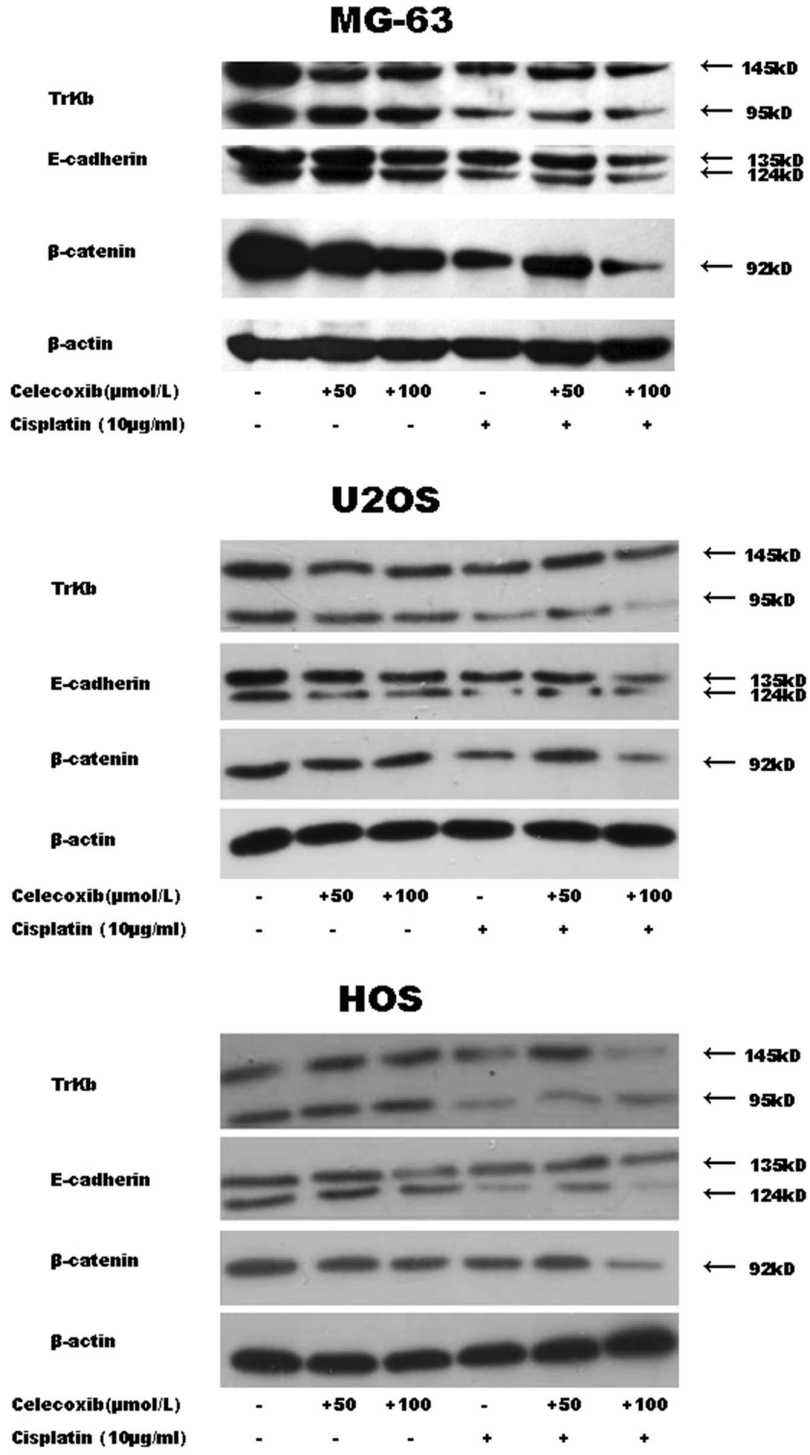
The down-regulations of Trkb, E-cadherin and β-catenin activation were detected in MG-63 cells treated with celecoxib (50, 100 µmol/l), cisplatin (10 µg/ml), and the combination for 48 h. Significant down-regulations of Trkb, E-cadherin and β-catenin activation were observed in MG-63 cells treated with cisplatin and celecoxib (100 µmol/l). However, the potentialization of celecoxib (50 µmol/l) and cisplatin was not demonstrated. β-actin was used as an equal loading control

We have reported that celecoxib, a cyclooxygenase-2 inhibitor, induces apoptosis in human osteosarcoma cell line MG-63 via down-regulation of PI3K/Akt. PI3K/Akt plays an essential role in the cell/extracellar matrix (ECM) and cell/cell adhesion. Lack of the correct adhesion, the adhesion-dependent signals will be interrupted, which will result in adhesion-related apoptosis: anoikis [18]. Trkb, E-cadherin and β-catenin play an important role in the cell/cell adhesion [19]. In this study we found the down-regulations of E-cadherin and β-catenin in MG-63, U2OS and HOS cells, as shown in Fig. 3. Significant down-regulations of Trkb, E-cadherin and β-catenin activation were observed in MG-63 cells treated with cisplatin and celecoxib (100 µmol/l).

### The effect of celecoxib and cisplatin on nuclear expression of β-catenin

**Fig. 4:**
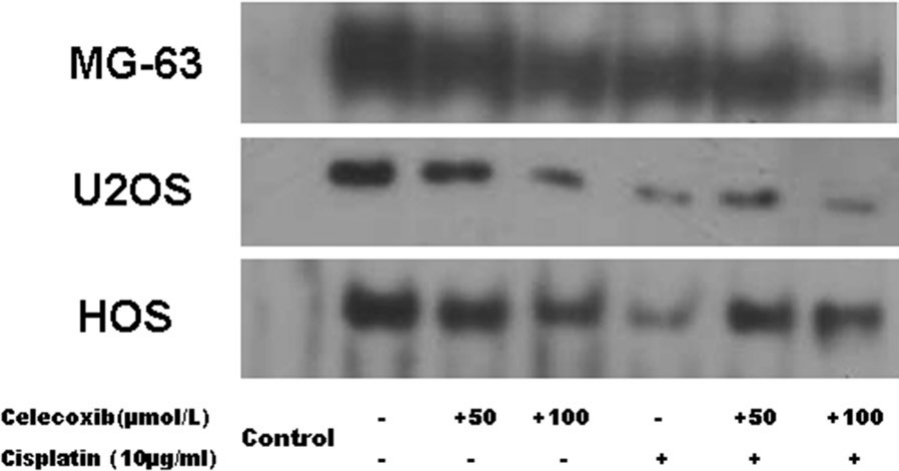
Constitutively active β-catenin binding activity was observed in nuclear extracts from MG-63, U2OS and HOS cells. In all cells, compared with untreated control, both celecoxib and cisplatin treatment obviously decreased β-catenin binding activity, meanwhile, significant down-regulation of β-catenin binding activity was displayed in MG-63 and U2OS cells

As show in Fig. 4, constitutively active nuclear expression of β-catenin binding activity was observed in nuclear extracts from MG-63, U2OS and HOS cells. In all cells, compared with untreated control, both celecoxib and cisplatin treatment obviously decreased β-catenin binding activity, meanwhile, significant down-regulation of β-catenin binding activity was displayed in MG-63 and U2OS cells. These results suggest that celecoxib not only down-regulates β-catenin binding activity but also sensitizes cisplatin effect, which may be responsible for enhanced inhibition of cell viability and inducing apoptosis by combination treatment.

### PI3K inhibition by wortmannin causing to the decease of pAkt and β-catenin

**Fig. 5:**
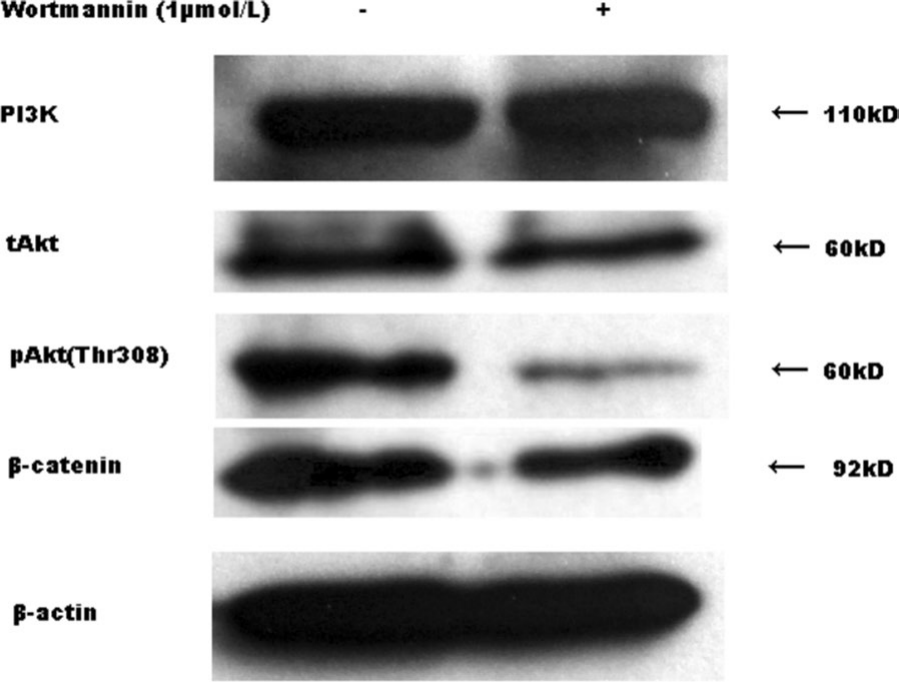
MG-63 cells were cultured in the presence (+) or absence (−) of 1 µmol/l Wortmannin for 48 h. The down-regulations of pAkt (Thr308) and β-catenin were detected, which were similar to cells treated with celecoxib

To investigate the role of PI3K in cells treated with celecoxib, we gave wortmannin (1 mmol/l) to cells for 48 h, but did not see downregulation of total PI3K and Akt, although downregulation of pAkt (Thr308) and β-catenin was detected (Fig. 5).

### A hypothetical model for PI3K and COX-2 pathway interaction

**Fig. 6:**
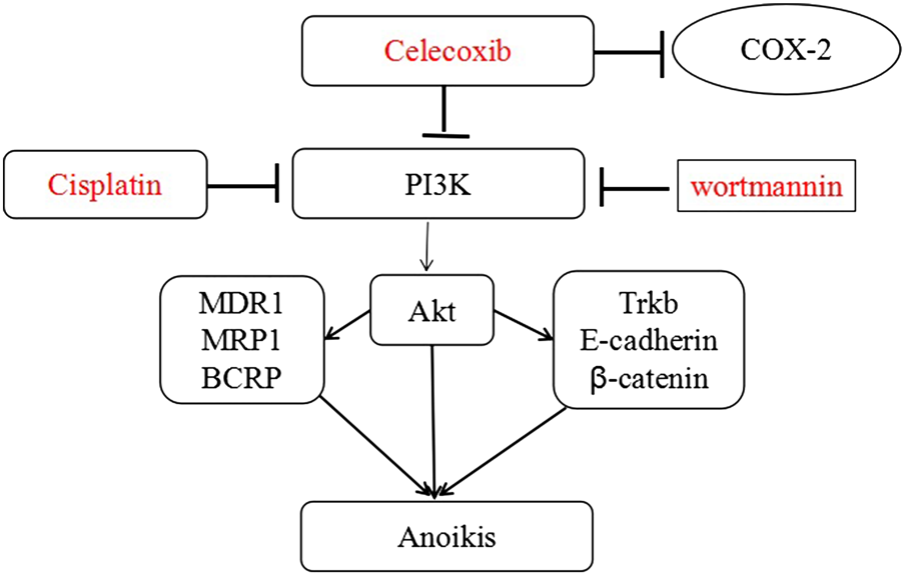
A hypothetical model for PI3K and COX-2 pathway interaction. Celecoxib down-regulates the expression of PI3K/Akt, which plays an essential role in the regulation of MDR1, MRP1, BCRP and Trkb, E-cadherin, β-catenin, eventually causing anoikis. PI3K activity negatively regulates expression of COX-2

Celecoxib down-regulates the expression of PI3K/Akt, which plays an essential role in the regulation of MDR1, MRP1, BCRP and Trkb, E-cadherin, β-catenin, eventually causing anoikis. PI3K negatively regulates the expression of COX-2. It demonstrate that celecoxib enhances the anoikis induction and cytotoxic effect of cisplatin (Fig. 6).

## Discussion

COX-2 is an inducible enzyme and associated with inflammatory diseases and carcinogenesis, which is suspected to promote angiogenesis and tissue invasion of tumors and resistance to apoptosis. COX-2 is also involved in drug resistance and poor prognosis of tumor. In the present study, it reveals that celecoxib and cisplatin inhibit cell growth via induction of apoptosis, and celecoxib potentiate the growth inhibition of cisplatin [5, 6]. In our study, a significant reduction in cell viability and apoptosis in MG-63 cells treated with the combination of celecoxib and cisplatin as compared with either celecoxib or cisplatin alone was observed.

Recently, a causal link between COX-2 and MDR-1 gene expression, implicated in cancer chemoresistance, has been demonstrated. Patel et al. [20] showed that the overexpression of COX-2 leads to increased P-gp expression and activity, and this effect is dependent on COX-2 activity. In addition, COX-2 inhibitor was able to block the COX-2-mediated increase in MDR1 expression and activity [20]. It has revealed that the expression of COX-2 and the downstream enzyme involved in PGE2 biosynthesis, mPGES1, was correlated with P-gp and Bcl-xL [21]. COX-2 overexpression induced increased MRP-1 expression resulting in chemoresistance to cisplatin [22]. Strong positive correlation between the expression of COX-2 and MDR1/P-gp was also observed in hepatocellular carcinoma, breast cancer and ovarian cancer [23–25]. In addition, COX-2 may be a factor that can regulate the expression of all three ABC transporters: MDR1/P-gp, MRP1 and BCRP [26].

Nonsteroidal anti-inflammatory drugs (NSAIDs) have been shown to induce apoptosis as well as potentiate the effect of chemotherapeutic agents including cisplatin. Celecoxib is a NSAIDs which is a specific inhibitor of COX-2. NSAIDs and COX-2 selective inhibitors have been demonstrated to overcome MDR in many cancers. It has been suggested that COX-inhibitors may sensitize cancer cells to chemotherapeutic drugs via inhibiting P-gp, MRP1 and BCRP, and enhance the effect of anticancer drugs [27]. In imatinib-resistant K562 cells, celecoxib can inhibit COX-2 and down-regulate MDR-1 expression through Akt/p-Akt signaling pathway [27]. Akt/protein kinase B (PKB) belongs to the downstream molecules of PI3K, which plays an important role in PGE2-EP4 pathway, as shown above. COX-2 inhibitors are known to inhibit the PI3K/Akt pathway [28]. We have confirmed that celecoxib induces apoptosis in human osteosarcoma cell line MG-63 via down-regulation of PI3K/Akt, as well as potentiates the effect of cisplatin [16]. In breast cancer, COX-2 inhibitors can also inhibit P-gp expression and function [29]. Celecoxib down-regulated the expression of MRP1 protein in human lung cancer, which was accompanied by increased accumulation and enhanced cytotoxicity of doxorubicin [30]. Ko et al. [31] demonstrated that celecoxib reverses BCRP- and MRP1-related drug resistance via the down-regulation of MRP1 and BCRP mRNA in squamous cell carcinoma. Therefore, COX-2 may play an important role in upregulation the expression of MDR1/P-gp, MRP1 and BCRP via the COX-2-PGE2-EP4-PI3K pathway. In addition, COX-2 inhibitors can reverse the effects of COX-2. And the mechanisms of COX-2 inhibitors regulate the transcription of the MDR still do further research.

PI3K/Akt plays a central role in regulation of adhesion [32]. In cell/cell attachment, β-catenin serves as a component of the cytoskeleton [19]. β-catenin associates with the intracellular tail of the intercellular adhesion molecule E-cadherin [33]. Through this association, β-catenin plays an important role in strong cell–cell adhesion as it links E-cadherin to the actin cytoskeleton through the protein α-catenin [34]. Another function of β-catenin is that maintaining cell-to-cell adhesion and mediating the Wnt/β-catenin signal transduction pathway, which plays pivotal roles in embryogenesis and in malignant transformation of cells [35]. It has reported that E-cadherin is lost from cell–cell contacts before the execution of apoptosis [36]. Activation of PI3K/Akt signaling has been shown to mediate survival signals triggered by the engagement of E-cadherin [37] and other classical cadherins [38, 39].

The present report suggests that β-catenin may also lie downstream of integrins. Several integrin-stimulated signaling pathways might lead to the induction of β-catenin signaling. One possible connection between integrins and β-catenin is the integrin-activated, antiapoptotic kinase PKB/Akt. PKB is known to inhibit the activity of glycogen synthase kinase 3-β, a serine kinase that functions directly to reduce β-catenin protein and signaling [40, 41]. It is possible that the result of these two inhibitory interactions is that activation of PKB by integrin signaling functions to positively activate β-catenin signaling. TrkB is a receptor tyrosine kinase that has brain-derived neurotrophic factor (BDNF) as one of its primary ligands. It has been established that BDNF binding to TrkB receptors results in a highly specific receptor autophosphorylation [42] and in turn activates the PI3K/Akt pathway [43].

In conclusion, MDR1, MRP1, BCRP and Trkb, E-cadherin, β-catenin were significantly downregulated in cells treated with the combination of celecoxib and cisplatin, and decreased β-catenin level was found in cells with wortmannin, a specific PI3K inhibitor. Therefore, celecoxib enhances anticancer effect of cisplatin and induces anoikis in osteosarcoma, which may be PI3K/Akt-dependent, and MDR and β-catenin-related. PI3K may be at the center of the celecoxib effects, which play an essential role in the regulation of MDR and anoikis.

## Abbreviations

COX: cyclooxygenase
PG: prostaglandin
NSAIDs: nonsteroidal anti-inflammatory drugs
CSC: cancer stem cell
ECM: extracellar matrix
MDR1/P-gp: multidrug resistance/P-glycoprotein
MRP1: multidrug resistance protein 1
BCRP: breast cancer resistance protein
